# The results of using a tendon autograft as a new rotator cable for patients with a massive rotator cuff tear: a technical note and comparative outcome analysis

**DOI:** 10.1186/s13018-020-1568-0

**Published:** 2020-02-12

**Authors:** Egbert J. D. Veen, Ronald L. Diercks, Ellie B. M. Landman, Cornelis T. Koorevaar

**Affiliations:** 1 Department of Orthopedics, University of Groningen, University Medical Center Groningen, Hanzeplein 1, Postbus 30.001, 9700 RB Groningen, The Netherlands; 2 Department of Orthopedic Surgery and Traumatology, Deventer Hospital, Deventer, The Netherlands

**Keywords:** Biceps tendon, Massive rotator cuff tear, Autograft, Arthroscopy

## Abstract

**Background:**

Several surgical reconstructive options are available to treat massive rotator cuff tears (MRCTs). The rotator cable has an important function and we evaluated the clinical result after arthroscopic reconstruction of the rotator cable with an autograft tendon.

**Methods:**

A prospective pilot study was performed with inclusion of four patients, average age of 64 years, with an irreparable MRCT. The patients underwent an arthroscopic reconstruction of the rotator cable with the use of the long head of biceps tendon autograft, except for one which was reconstructed with a hamstring tendon. Pre- and postsurgically, the Constant-Murley Score (CMS), Western Ontario Rotator Cuff Index (WORC), Simple Shoulder Test (SST), visual analog scale (VAS) scores, and an MRI was performed. Clinical results of the study group were compared with clinical results of comparable cohort of patients with a MRCT, treated non-operatively with physiotherapy.

**Results:**

The CMS score increased after surgery in three of the four patients. The improvement of CMS score was comparable to the improvement of the CMS score encountered in a comparable cohort. The MRI at 12 months follow-up showed that the reconstructed rotator cable was disintegrated in all patients and the rotator cuff was detached and retracted.

**Conclusions:**

In our pilot study, arthroscopic reconstruction of the rotator cable using a tendon autograft failed over time and showed no clinical benefit in comparison to the non-operative treatment with physiotherapy.

**Trial registration:**

The regional Medical Ethical Committee (Zwolle) gave approval at 14th of October 2016 and assigned no. 16.06100.

## Introduction

Massive rotator cuff tears (MRCTs) are defined as cuff tears involving two or more cuff tendons or a retraction of ≥ 5cm [1]. Patients frequently present with pain and loss of function (pseudoparalysis), sometimes after minimal trauma. A MRCT can have a huge impact on daily life and eventually could lead to a cuff arthropathy. Variable success rates are seen after primary cuff repair in small-to-medium-sized tears with retear rates between 14 and 25% [2]. If the repair is successful and the cuff has healed after surgery, patients have a better outcome compared with patients after conservative or failed surgical therapy [3, 4]. MRCT defects cannot be easily closed, and there is a rate of retears reaching up to 94% [5, 6]. Several surgical reconstructive options for MRCTs are available, each with its own reported advantages and complications [7]. Patches and grafts are expensive and can lead to rejection. Another technique is superior capsular reconstruction to treat these lesions [8]. Initial results for reconstruction of the superior capsule seem promising, although long-term results are awaited. Reversed shoulder arthroplasties and tendon transfers are alternative options, yet these are major surgeries with considerable morbidity [9].

A tenotomy or tenodesis of the long head of the biceps tendon is often performed as part of rotator cuff surgery. This offers the possibility of using the biceps tendon as a graft. Different studies used this tendon as a free graft or leaving the distal or proximal attachment intact, reporting significant improvement of function [10]. In the native shoulder, the rotator cable is a thickening in the rotator cuff that serves as a primary load-bearing structure between the rotator cuff and the humerus and functions as a tension bridge. Recent studies show the clinical and biomechanical importance of this structure [11, 12].

We developed a technique to treat MRCTs by reconstructing the rotator cable with a long head of biceps tendon autograft [13]. But a hamstring tendon is also suitable. This technique represents a reconstruction with several advantages: use of an autograft, ease of harvesting, no graft reactions, fixation of cuff to reconstructed rotator cable instead of footprint, and potential prevention of cuff arthropathy.

The aim of this study is to evaluate the clinical and radiological result after arthroscopic reconstruction of the rotator cuff cable with an autograft tendon in patients with irreparable massive rotator cuff tears.

## Methods

### Indications

A prospective pilot study was conducted between February and December 2016. Patients aged 50 or older diagnosed with an irreparable MRCT (2–3 tendon tears, Patte stage 3 retraction), based on MRI, were included [14]. At physical examination, all had impaired abduction and decreased strength of the infraspinatus and supraspinatus muscles. All had undergone conservative treatment of at least 3 months consisting of physiotherapy and/or subacromial injections, without the expected effect. Exclusion criteria were as follows: symptomatic glenohumeral or acromioclavicular osteoarthritis, rheumatoid arthritis, previous surgery on the same shoulder or arm, and cognitive or linguistic issues. After 1 year of inclusion, the clinical results of this new operative technique were evaluated. Four patients were included in this year. The local institutional review board approved this study (no. 16.06100), and all patients gave informed consent. The study was done according to the ethical standards of the 1964 Helsinki Declaration and its later amendments.

Primary outcome was the Constant-Murley Score (CMS). We looked secondarily at the Simple Shoulder Test (SST) [15], Western Ontario Rotator Cuff Index (WORC) [16], and visual analog scale (VAS) [17] on pain, disability, and patient satisfaction. Scores were taken preoperatively at 3, 6, and 12 months.

Prior to surgery and 12 months postoperatively, an MRI scan was performed and the rotator cuff retraction and fatty infiltration were graded with Patte and Fuchs scores [14, 18]. Extension of the tear and number of torn tendons were also noted. Evaluation was done by an experienced musculoskeletal radiologist.

The results were compared with a cohort of patients with a MRCT, Patte stage 3, treated non-operatively with physiotherapy. These patients were selected from a previous RCT performed at our institution, comparing surgical rotator cuff repair with conservative treatment for degenerative rotator cuff tears [3]. The conservative protocol has been described previously, and scores were collected with the same interval during 12 months [3]. From the patients treated non-operatively with physiotherapy, eight patients with a MRCT had a Patte stage 3 and were included; in five patients, all follow-up data were available and used as a comparable cohort.

### Surgical procedure

This technique was first tested on cadavers to assess feasibility and detect any pitfalls. All surgeries were performed by one shoulder surgeon (CK). The surgical technique was extensively described in a previous report [13].

Patients were operated in the beach chair position after an interscalene block of the brachial plexus and general anesthesia. The standard procedure started by introducing the scope through the posterior portal. After confirming the diagnosis of MRCT, the intra-articular portion of the biceps was tenotomized just distally of the insertion on the superior labrum. Next, the tendon was harvested through a small anterolateral incision at the bicipital sulcus. In case this tendon was degenerated, a hamstring autograft was used.

### Cuff preparation and mobilization

The subacromial space was inspected, and a bursectomy was performed through a lateral portal to create a clear view of the remnants of the rotator cuff. An extensive release of the cuff was performed subacromially and between the cuff and the superior labrum with close attention to the suprascapular nerve.

### Biceps graft fixation

**Fig. 1:**
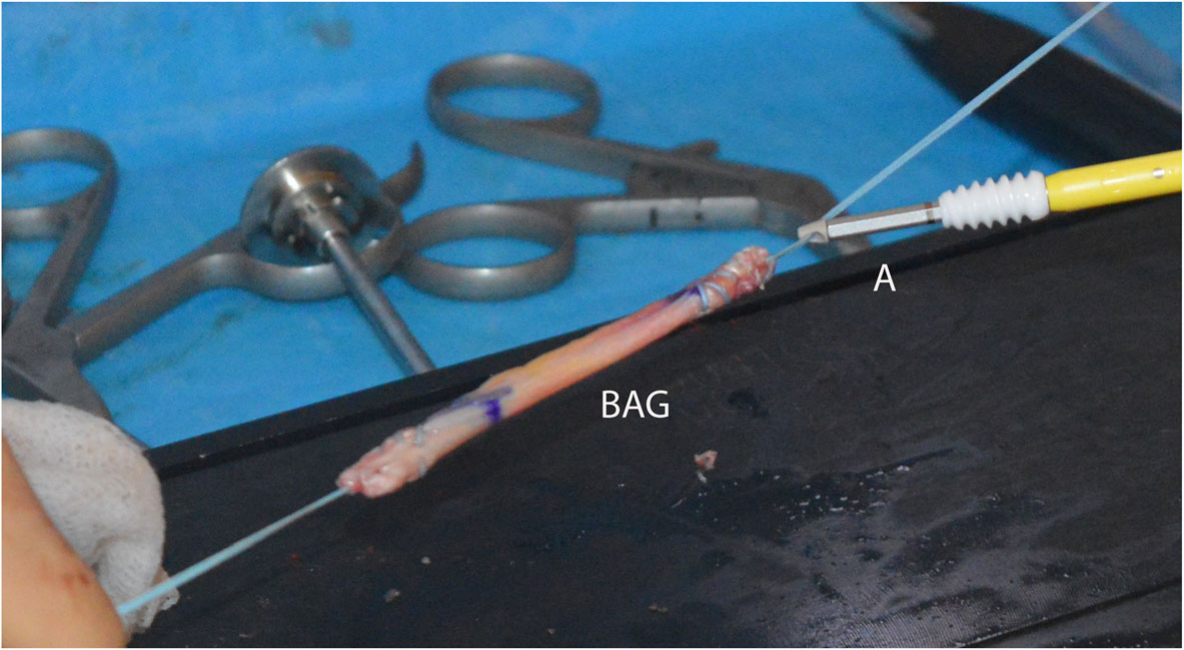
Final preparation of the biceps autograft with the anchor attached to the graft. BAG: biceps autograft, a: tenodesis biocomposite anchor (7 mm x 19.1 mm)

**Fig. 2:**
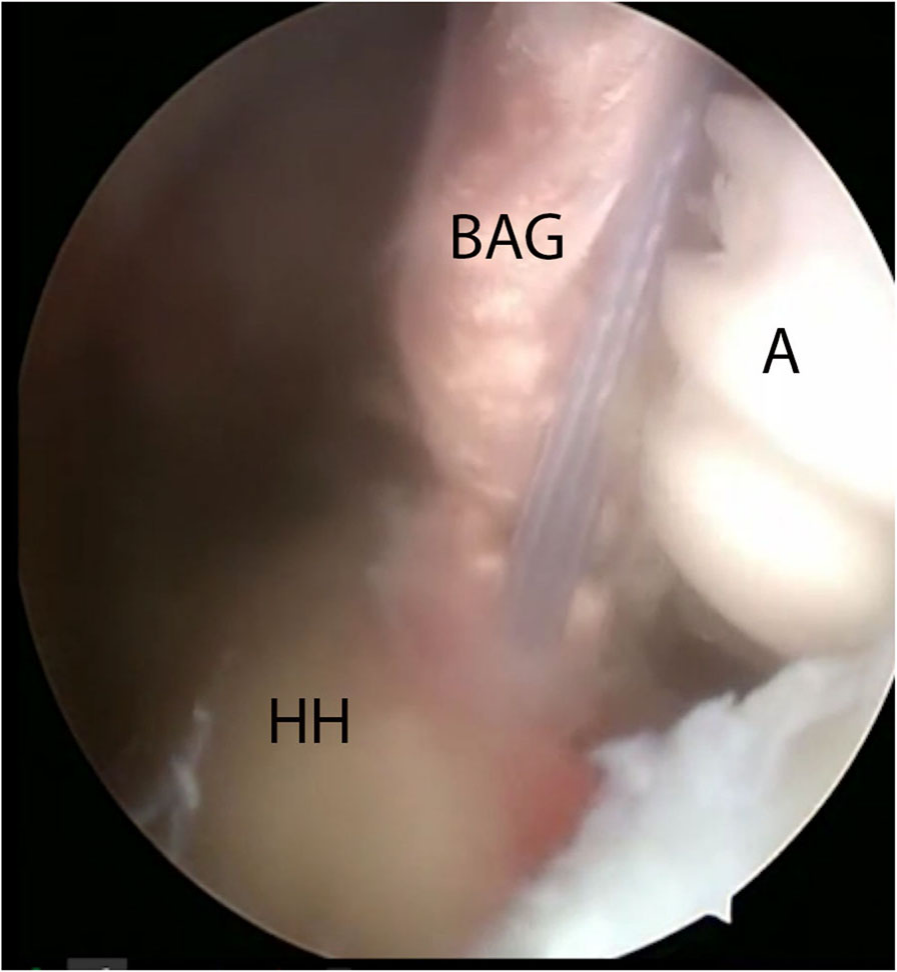
Insertion of anchor with biceps autograft attached into the humeral head after drilling. Seen from a posterior portal. HH: humeral head, BAG: biceps autograft, A: anchor

The tendon was prepared and marked, with 20 mm tendon left on each side to be inserted into the humeral bone with biotenodesis screws (tenodesis biocomposite anchors, 7 × 19.1 mm) (Fig. 1). An additional posterolateral portal was made to drill a hole for the posterior screw with a diameter equal to the tendon, approximately in the middle of the footprint of the infraspinatus on the greater tubercle. With the biceps autograft under tension, this posterior screw was tightly inserted until the pre-marked portion of the biceps tendon (Fig. 2). After positioning of the graft, a drill hole was made in the bicipital groove at the superior part of the lesser tubercle.

### Final rotator cuff repair to rotator cable

**Fig. 3:**
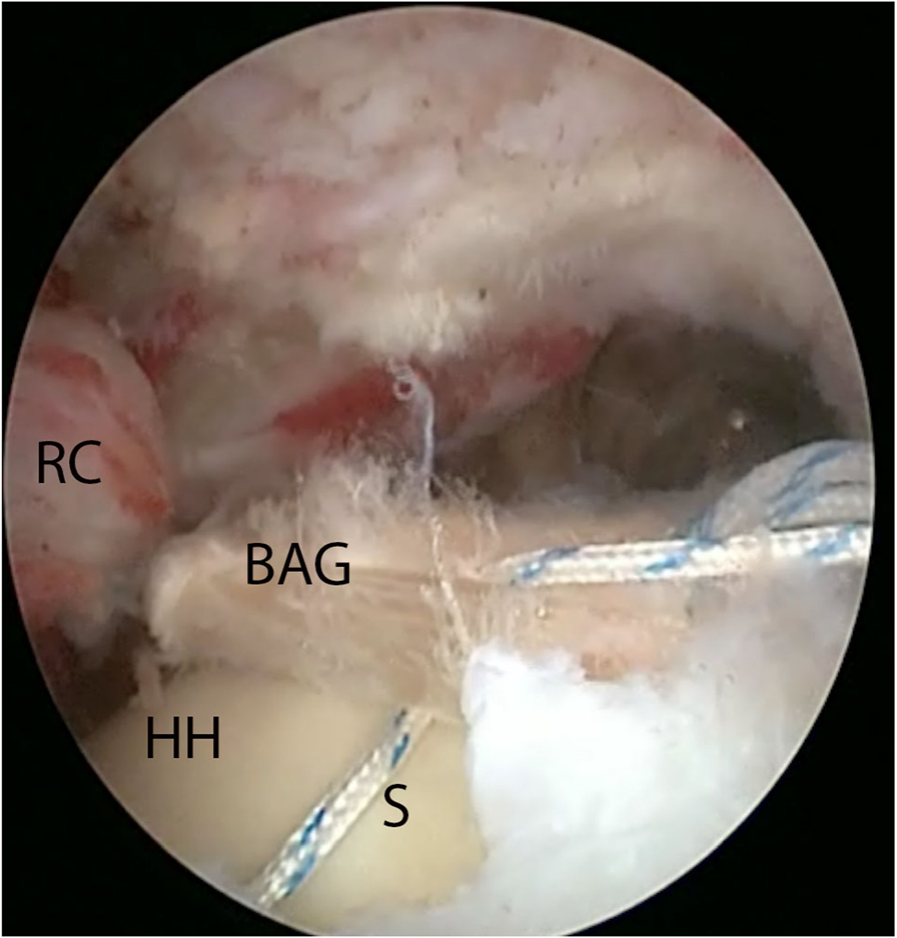
Attaching the cuff to the new reconstructed rotator cable using sutures. Seen from a posterior portal. RC: rotator cuff, HH: humeral head, BAG: biceps autograft, S: suture

The final step was the actual cuff repair. The sutures were passed transversely around and through the biceps tendon autograft (Fig. 3). The medial part of the sutures was passed through the infraspinatus and supraspinatus tendons with a suture-passing device. An average of five sutures were needed in order to bring the rotator cuff to the tendon autograft.

### Postoperative protocol

The shoulder was immobilized for 6 weeks, during which passive movements were allowed under the guidance of a physiotherapist. Afterwards, further guided active mobilization was started.

### Statistical analysis

Patient characteristics are expressed descriptively. The SPSS statistical software (version 20.0; IBM, Armonk, NY, USA) was used for data compilation and statistical analyses. Only descriptive statistics were considered suitable considering the number of patients in each group. Because of the pilot study design, a sample size calculation was not feasible.

## Results

A total of four patients were included in this study. Patient characteristics are depicted in Table 1. Mean age of the study population was 64 years (61–67 years), all with their dominant side affected, and one was a smoker. In all patients, a massive cuff tear of the supraspinatus and infraspinatus tendon was present with a Patte stage 3 retraction; in one patient, a partial subscapularis tendon tear was found and repaired. Fatty infiltration on the postoperative MRI scan differed between Fuchs stages 2 to 3 on the preoperative MRI. One patient discontinued the study 9 months after surgery because of lack of postsurgical improvement, and a reversed shoulder prosthesis was inserted. The final scores of this patient were considered as an endpoint.

**Table 1:** Patient characteristics

Patient	Age (years)	Sex	Smoker	MRI, no. of ruptured tendons	MRI Patte	MRI Fuchs	Additional surgery
Group 1							
1	61	Female	Yes	2	3	3	
2	67	Male	No	2	3	2	
3	64	Male	No	3	3	3	Hamstring autograft, subscapularis repair
4	62	Male	No	2	3	2	
Group 2							
1	72	Female		2	3	3	Non-surgical treatment
2	63	Female		1	3	2	Non-surgical treatment
3	62	Male		2	3	3	Non-surgical treatment
4	72	Female		3	3	–	Non-surgical treatment
5	57	Female		2	3	3	Non-surgical treatment

Group 1, surgical treatment, group 2, non-surgical treatment

For comparison, a similar group of patients who were treated conservatively was selected. This comparable cohort consisted of five patients, mean age was 65 years (57–72 years), and more women were present in this group. All patients had a massive cuff tear of the supraspinatus and infraspinatus tendon with a Patte stage 3 retraction; in one patient only, the supraspinatus was torn and was treated using physiotherapy.

**Fig. 4:**
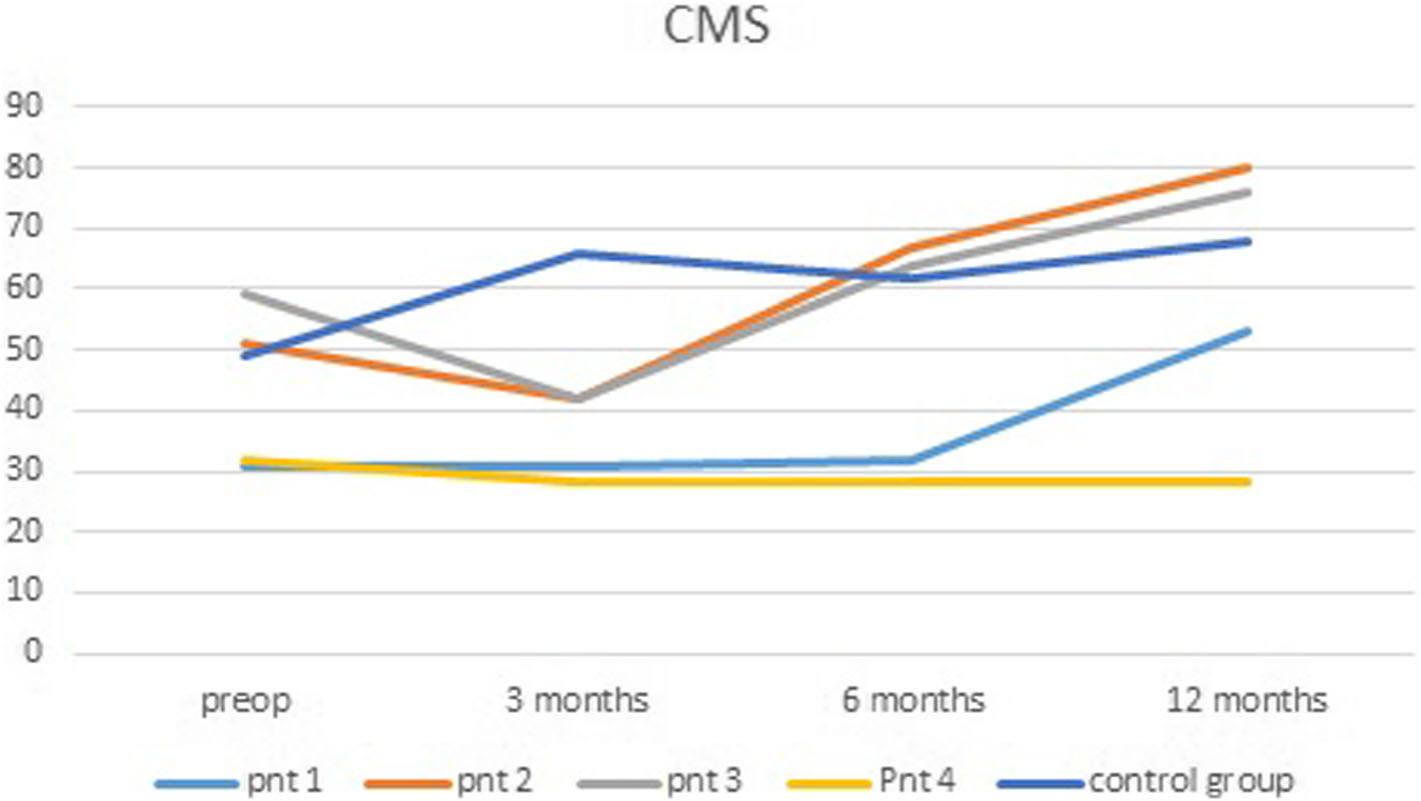
Total CMS scores per patient

Table 2 shows the clinical outcome of the study group after surgery and the group treated with physiotherapy. Total CMS score increased significantly after surgery in three of the four patients, with a mean improvement of 26.4 points after 12 months (*p* = 0.023) (Fig. 4). The WORC index, depicted as a percentage of a normal score, showed a mean improvement of 5 points (*p* = 0.191). SST, VAS pain, VAS disability, and VAS satisfaction scores also improved in these patients. All except for patient 4 showed improvement on the scores. In all patients, an MRI was performed 12 months after surgery. In all patients, the reconstructed rotator cable had disintegrated and the rotator cuff was detached from the rotator cable and retracted. Fatty infiltration of the infraspinatus muscle before surgery was grade 2 in two patients and grade 3 in two patients. At follow-up, all patients had fatty infiltration of the infraspinatus muscle grade 3 on MRI. The partial subscapular repair was healed on MRI in patient 3. None of the patients had any signs of osteoarthritis on MRI before or after surgery.

**Table 2:** Outcome scores in mean (range)

	Preop		3 months		6 months		12 months^a^	
	Group 1	Group 2	Group 1	Group 2	Group 1	Group 2	Group 1	Group 2
CMS pain	6.3 (0–10)	7.0 (0–15)	7.5 (5–10)	13.7 (10–15)	10.0 (5–10)	10.0(5–15)	13.3 (10–15)	11.0 (0–15)
CMS activity	7.0 (4–12)	10.4 (4–14)	5.0 (4–6)	16.5 (15–19)	9.3 (4–15)	14.8(12–20)	16.0 (10–20)	16.4 (8–20)
CMS mobility	17.5 (10–24)	23.6 (8–36)	10.0 (8–12)	26.0 (20–30)	14.5 (8–20)	27.2(22–34)	20.6 (18–26)	30.8 (20–36)
CMS strength	12.5 (5–25)	9.46 (2–19)	12.5 (5–20)	9.65 (8–12)	14.3 (5–22)	10.4(4–18)	19.7 (10–25)	9.88 (2–17)
CMS total	43.3 (31–32)	49.0 (26–73)	35.0 (28–42)	65.9 (61–73)	46.5 (28–67)	62.0(51–69)	69.7 (53–80)	68.1 (30–81)
SST	5.5 (1–8)	–	4.75 (1–8)	–	5.3 (2–7)	–	6.3 (6–7)	–
WORC	55.3 (36–52)	–	43.3 (39–53)	–	47.7 (32–60)	–	60.3 (49–67)	–
VAS pain	45.0 (30–70)	60 (30–80)	56.3 (20–80)	27 (20–50)	37.5 (10–65)	38(20–60)	16.7 (10–20)	26 (10–80)
VAS disability	53.3 (8–80)	54 (20–10)	65.0 (40–85)	35 (30–40)	46.3 (20–70)	42 (30–60)	40.0 (15–70)	30 (10–80)
VAS satisfaction	–	–	46.3 (40–50)	–	43.8 (5–60)	–	63.4 (45–85)	–

Group 1, surgical treatment; group 2, non-surgical treatment

WORC depicted as a percentage of normal/maximum score

^a^Results of three out of four patients

Compared with the patients who were treated conservatively, the CMS and VAS pain and disability scores of the surgical group were similar before treatment and at 12 months follow-up. At 3 and 6 months after surgery, the clinical results were inferior to the non-operative group.

## Discussion

Clinical and radiological results after tendon autograft for reconstruction of the rotator cable were collected in four patients with a MRCT. On the MRI scan, 1 year after surgery, the rotator cable reconstruction disintegrated and failed; clinical results were not superior to the results after non-operative treatment with physiotherapy. Because of disappointing preliminary results and a vanishing reconstructed rotator cable on MRI during 1 year of inclusion, no further patients were included. When developing this new surgical procedure, we hypothesized that the procedure might have several advantages. This technique results in a biomechanical reconstruction leading to transporting force couples from the rotator cuff to the humeral head. As the remaining rotator cuff was attached to the rotator cable and not to the footprint, we were able to reattach the rotator cuff to the cable in irreparable cuff tears. With this technique, tension on the massive cuff tear gap was minimized by medializing the new rotator cable. The technique represents a reconstruction with the use of an autograft. The biceps tendon is easy to harvest and gives no graft reactions. In other studies, the biceps tendon was used as an augmentation of the rotator cuff with good clinical results. Although we observed improved clinical results in three out of four patients 1 year after surgery with the CMS and WORC exceeding the minimal important clinical difference of 10.4 points [19] and 282.6 points [20] respectively, MRI showed failure of the reconstructed rotator cable in all patients.

While performing the index operation, we used margin convergence to close the cuff partially to the reconstructed rotator cable, but on MRI scan at follow-up, the cuff was re-torn and retracted compared with the preoperative situation. The improvement in pain and function could not be attributed to the reconstruction of the rotator cable as this construct failed over time [21]. The clinical improvement over time in the surgical group was similar to the improvement noted in the control group, treated non-operatively with physiotherapy. In two randomized controlled studies about cuff repair versus conservative treatment of degenerative rotator cuff tears, good clinical improvement was also found after conservative treatment. These studies showed a mean improvement of the CMS of 16.8 points after 1 year [3] and 18.4 points after 2 years [22] following conservative treatment. In both studies, this was not significantly different from the surgically treated patients. In our control group, a mean improvement of the CMS of 19.1 points was found after 1 year.

The rotator cuff showed fatty infiltration (grade 3 in two out of four patients). This may have resulted in low healing capacity and eventually a detachment of the rotator cuff from the reconstructed rotator cable on the control MRI. Previous studies also show that this is a predictor for worse outcomes in cuff repairs [23], even in cases of successful repair [24]. Reconstruction of the rotator cable failed over time in all patients. The biceps tendon may have been degenerative, thus preventing ingrowth, although histological analyses show that a degenerated biceps tendon is still rich in collagen [25]. In one patient, a hamstring graft was used because of a missing long head of biceps during surgery. This may be a bias confounding the outcome. The study group is small and consisted only of four patients. In our study protocol, we decided to include patients for this new technique for 1 year, and then evaluate the clinical and radiological results. Because the construction failed in all patients, we decided to end this pilot study. There is a possibility that this technique might be successful in some patients when a larger group of patients would be operated. Comparing the results of our study with other studies using a biceps tendon graft for rotator cuff repair, a superior outcome was found when the biceps tendon was used for augmentation with an improvement of 44.1 points on the CMS by Cho et al. [26]. Also, the long-term outcome scores of the latissimus dorsi transfer as treatment for massive rotator cuff tears were better, with an increase of 27 points on the CMS [27].

Treating MRCTs remains a significant challenge for the clinicians. Several surgical options are available for patients with a MRCT if conservative treatment fail: debridement, long head of biceps tenotomy, partial repair, rotator cuff advancement, bridging graft repair, superior capsular reconstruction, subacromial spacer, and reverse total shoulder arthroplasty. The variability in patient characteristics, co-interventions, outcome reporting, and length of follow-up in studies on MRCTs complicates sound comparison of treatments [28]. Joint preserving procedures are preferably used in young patients; in the older population, reversed shoulder arthroplasty or maybe the subacromial balloon spacer might be indicated. The superior capsular reconstruction has become a popular surgical technique. The short-term results of superior capsular reconstruction show consistent improvement in shoulder functionality and pain reduction. However, on longer follow-up, decreased acromiohumeral intervals indicate dermal allograft elongation and persistent superior migration of the humerus [29]. Placement of the subacromial balloon spacer is a minimally invasive, technically simple procedure with favorable patient-reported outcomes at limited short-term follow-up. However, inherent methodological limitations and patient heterogeneity between studies using the subacromial spacer may impair the ability to fully characterize the long-term efficacy, particularly relative to other potential surgical options [30, 31].

## Conclusions

In conclusion, the arthroscopic reconstruction of the rotator cable using a tendon autograft failed over time in this pilot study, and showed no clinical benefit in comparison with the non-operative treatment with physiotherapy in patients with a MRCT. We therefore cannot recommend using this surgical procedure to treat patients with a massive rotator cuff tear.

## Data Availability

The datasets used and/or analyzed during the current study are available from the corresponding author on reasonable request.

## Abbreviations

CMS: Constant-Murley Score
MRCT: Massive rotator cuff tear
MRI: Magnetic resonance imaging
SST: Simple Shoulder Test
VAS: Visual analog scale
WORC: Western Ontario Rotator Cuff
